# Sonicated *Bordetella bronchiseptica* Bacterin Can Protect Dendritic Cells from Differential Cytotoxicity Caused by Doxorubicin and Vincristine and Enhance Their Antigen-Presenting Capability

**DOI:** 10.3390/cimb44070213

**Published:** 2022-07-06

**Authors:** Ji Yun Sung, Hong-Gu Joo

**Affiliations:** Laboratory of Veterinary Pharmacology, College of Veterinary Medicine, Jeju National University, Jeju 63243, Korea; sungjy1112@gmail.com

**Keywords:** doxorubicin, vincristine, dendritic cell, sonicated *Bordetella bronchiseptica*, protective effects

## Abstract

Doxorubicin (DOX) and vincristine (VC) are anti-cancer drugs commonly used for lymphoma in veterinary and human medicine. However, there are several side effects caused by these drugs. In this study, the protective effects of sonicated *Bordetella bronchiseptica* bacterin (sBb) on dendritic cells (DCs) damaged by two anti-cancer drugs were investigated. DCs play important roles in the innate and adaptive immunity of hosts, especially activating T cells that can suppress tumor growth. The metabolic activity of DCs significantly increased after the treatment with sBb compared to that of control DCs. In addition, there was a marked change in mitochondrial integrity between DOX-treated DC and DOX + sBb-treated DCs. Flow cytometric analysis also demonstrated that sBb upregulated the expression of the surface markers of DCs, particularly CD54. In mixed lymphocyte responses, sBb significantly increased the antigen-presenting capability of DCs. In particular, sBb increased the capability of control DCs by approximately 150% and that of VC-treated DCs by 221%. These results suggest that sBb can be used as a potential immunostimulatory agent to protect DCs from anti-cancer drug-induced damage and provide fundamental information about using a combination of DCs and vincristine in immunotherapy.

## 1. Introduction

Dendritic cells (DCs) play important roles in the innate and adaptive immunity of hosts. Although DCs comprise only a small population within immune cells, their influence on the initiation of antigen-specific immunity is prominent [1,2]. In the tumor microenvironment, DCs acquire and process tumor-associated antigens, and antigenic peptides are presented in the context of major histocompatibility complex (MHC) molecules and induce anti-tumor immune responses [2]. However, the function of DCs in tumors can be affected by multiple tumor-derived immunosuppressive factors and anti-cancer drugs [2,3,4]. Considering that DC immunotherapy is performed together with chemotherapy [5,6], the evaluation of anti-cancer drug damage for DCs and the study of cytoprotective methods for DCs are essential for the development of new cancer therapies [7].

Doxorubicin (DOX) and vincristine (VC) are anti-cancer drugs commonly used for the treatment of lymphoma in veterinary and human medicine [8,9]. DOX functions by binding to DNA-associated enzymes, intercalating with DNA base pairs, and targeting various molecular targets to produce cytotoxic effects [10], whereas VC acts as an anti-microtubule agent that blocks mitosis, arresting cells in the metaphase [11,12]. However, DOX can potentially cause severe cardiotoxicity, specifically cardiomyopathy, typhlitis, and erythema, among others, when used in cumulative doses [13,14]. DOX-induced cardiotoxicity is caused through Toll-like receptors, biomolecules that are expressed on the surfaces of cardiomyocytes and cardiovascular endothelial cells [15]. Furthermore, DOX shows toxicity to DCs at a concentration of 0.1–50 μM [16]. VC mainly induces chemotherapy-induced peripheral neuropathy, hyponatremia, constipation, and hair loss [17]. In one study, VC-induced toxicity against DCs was evaluated in a range of concentration of 0.1–50 nM [3]. Interestingly, noncytotoxic concentrations of VC (1 nM) was shown to up-regulate CD40, a maturation molecule of DCs [3].

In a previous study, we found that sonicated *Bordetella bronchiseptica* bacterin (sBb) can up-regulate the function of DCs, including increasing the marker expression and antigen-presenting capability of DCs, which means that it can be used as a potential adjuvant to enhance the efficacy of vaccines [18]. In this study, we evaluated the cytotoxic damage caused by two anti-cancer drugs to DCs and investigated the cytoprotective effects of sBb on DCs treated with the anti-cancer drugs.

## 2. Materials and Methods

### 2.1. Animals and Reagents

C57BL/6 and Balb/c mice were purchased from Samtako (Korea) and housed at the animal facility of Jeju National University. In this study, 7- to 12-week-old female mice were used for experiments. Animal experiments in this study were performed in accordance with the Institutional Guidelines for Animal Use and Care of Jeju National University (2021-0015). DOX and VC were purchased from Sigma-Aldrich (St. Louis, MO, USA) and dissolved in dimethyl sulfoxide and phosphate buffer solution (PBS), respectively. *Bordetella bronchiseptica* in PBS was sonicated under conditions of amplitude 60% for 3 cycles of 30 sec with a 30 sec interval using a Q125 sonicator (Qsonica, Newtown, CT, USA), then the amount of protein was measured by the Bradford assay and the proteinsBb was stored in a −20 °C freezer.

### 2.2. Preparation of DCs

Bone marrow cells were harvested from a femur and tibia from each C57BL/6 mouse. The cells were cultured in 6-well plates in RPMI 1640 medium containing 5% fetal bovine serum, 100 U/mL penicillin-streptomycin, 10 ng/mL mouse recombinant granulocyte macrophage-colony stimulating factor (PeproTech, Rocky Hill, NJ, USA), and recombinant mouse IL-4 (PeproTech). Culture medium was replaced with new medium every two days.

### 2.3. Measurement of the Metabolic Activity and Viability of DCs

The metabolic activity of DCs was evaluated using a *3-(4,5-dimethylthiazol-2-yl)-2,5-diphenyltetrazolium bromide* (MTT) (Sigma-Aldrich, St. Louis, MO, USA) assay. DCs were cultured in 96-well plates and treated with DOX, VC, and sBb for 3 days. After the treatment, MTT solution was added into wells at a concentration of 0.5 mg/mL for 4 h, and then 100 μL/well of 10% SDS solution was added to dissolve formazan for 2 h. The optical density was measured at 570 nm with a microplate reader (Multiskan FC; Thermo Fisher Scientific, Waltham, MA, USA). To measure viability, trypan blue exclusion assay was performed [19]. The number of viable and dead cells were counted, and the viability was calculated.

### 2.4. Flow Cytometry Analysis

To analyze the function of mitochondria, we measured the mitochondrial membrane potential (MMP) of cells. The treated DCs were stained with Rhodamine 123 solution at a concentration of 0.25 μg/mL in the dark for 30 min. To measure the expression of activation- or maturation-related surface markers, DCs were treated with DOX, VC, and sBb in 6-well plates for 3 days. The treated cells were stained with fluorescein isothiocyanate (FITC)-labeled anti-mouse MHC class II (I-A^b^), phycoerythrin (PE)-labeled anti-mouse CD40, PerCP Cy5.5-labeled anti-mouse CD54, and allophycocyanin (APC)-labeled anti-mouse CD86 (all from BioLegend, San Diego, CA, USA) at 4 °C for 30 min. TruStain FcX^TM^ (anti-mouse CD16/32; clone 93; BioLegend) was used for FC receptor blockade. Subsequently, all stained cells were analyzed using flow cytometry (LSR Fortessa^TM^, BD, Franklin Lakes, NJ, USA) and the FlowJo software (BD Biosciences, Franklin Lakes, NJ, USA).

### 2.5. Mixed Lymphocyte Reaction

To investigate the antigen-presenting capability of DCs, a mixed lymphocyte reaction was performed. DCs were treated with DOX, VC, and sBb for 2 days prior to co-culture. Allogeneic spleen cells harvested from Balb/c mice and the treated DCs were co-cultured at concentrations of 2 × 10^6^ cells/mL and 1 × 10^5^ cells/mL, respectively, in 96-well plates for 5 days. Before co-culture, DCs were treated with mitomycin C (Sigma-Aldrich) at a concentration of 50 μg/mL for 30 min to prevent their activation during co-culture. Subsequently, Cell Counting Kit-8 (Dojindo Molecular Tech, Mashiki, Japan) solution was added for 4 h. The optical density was measured at 450 nm with a microplate reader (Multiskan FC; Thermo Fisher Scientific).

### 2.6. Cytokine Production

To investigate the function of DCs, cells were treated with DOX, VC, and sBb in 96-well plates at 37 °C for 3 days. Then, the supernatants of treated cells were used for enzyme-linked immunosorbent (ELISA) assay. TNF-α, IL-6 (BioLegend), IL-1β, and IL-12 (Invitrogen, Waltham, MA, USA) ELISA kits were used according to the manufacturer’s instructions.

### 2.7. Statistical Analysis

Statistical analyses were performed with Prism 9 (GraphPad Software, San Diego, CA, USA). Data are expressed as mean ± standard deviation in the figures. All experiments were performed more than three times. A *p*-value of <0.05 was considered significant.

## 3. Results

### 3.1. The Effect of sBb on Cell Metabolic Activity of DCs

**Figure 1:**
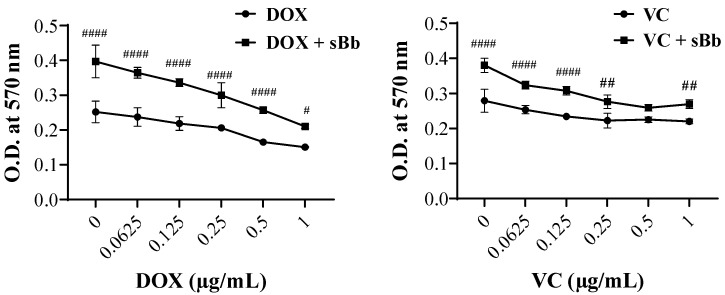
Metabolic activity of DCs treated with DOX, VC, and sBb. The DCs were treated with DOX, VC, and sBb for 3 days, then the MTT assay was performed. The optical density was measured at 570 nm with a microplate reader. #, ##, and #### indicate *p* < 0.05, 0.01, and 0.0001 compared to the metabolic activity of DCs in the absence or presence of sBb, respectively.

To evaluate protective effects of sBb, an MTT assay was performed. The DCs were treated with various concentrations of DOX and VC and 0.1 µg/mL sBb (Figure 1). The metabolic activity of DCs treated with both DOX and VC and sBb significantly increased compared to that of DCs treated with DOX and VC alone. Based on these results, we decided to use concentrations of 0.25 and 0.5 μg/mL for both DOX and VC to further investigate the effects of sBb on DCs.

### 3.2. Increment of DC Viability Damaged by Anti-Cancer Drugs

**Figure 2:**
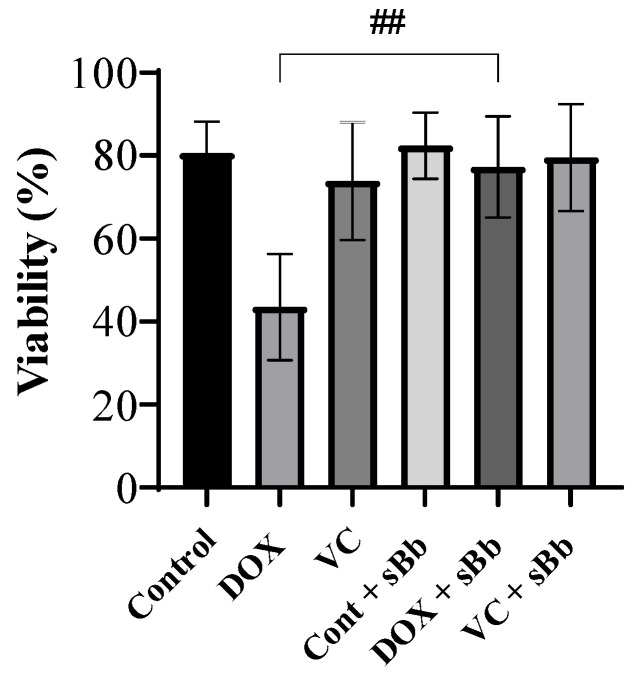
sBb increases the viability of DCs treated with DOX. The DCs were treated with 0.25 μg/mL DOX, 0.25 μg/mL VC and 0.1 μg/mL sBb for 2 days, then trypan blue exclusion assay was performed. The number of viable and dead cells were counted, and the viability rate was calculated. ## indicates *p* < 0.01 compared to the viability of DCs in the absence or presence of sBb.

To assess the viability of DCs treated with DOX, VC, and sBb, a trypan blue exclusion assay was performed. The number of viable and dead cells were counted and the viability was calculated (Figure 2). DCs treated with DOX alone presented the lowest viability. However, when the DOX + sBb treatment was applied, the viability of the cells increased significantly.

### 3.3. sBb Increases the Mitochondrial Function of DOX-Treated DCs

**Figure 3:**
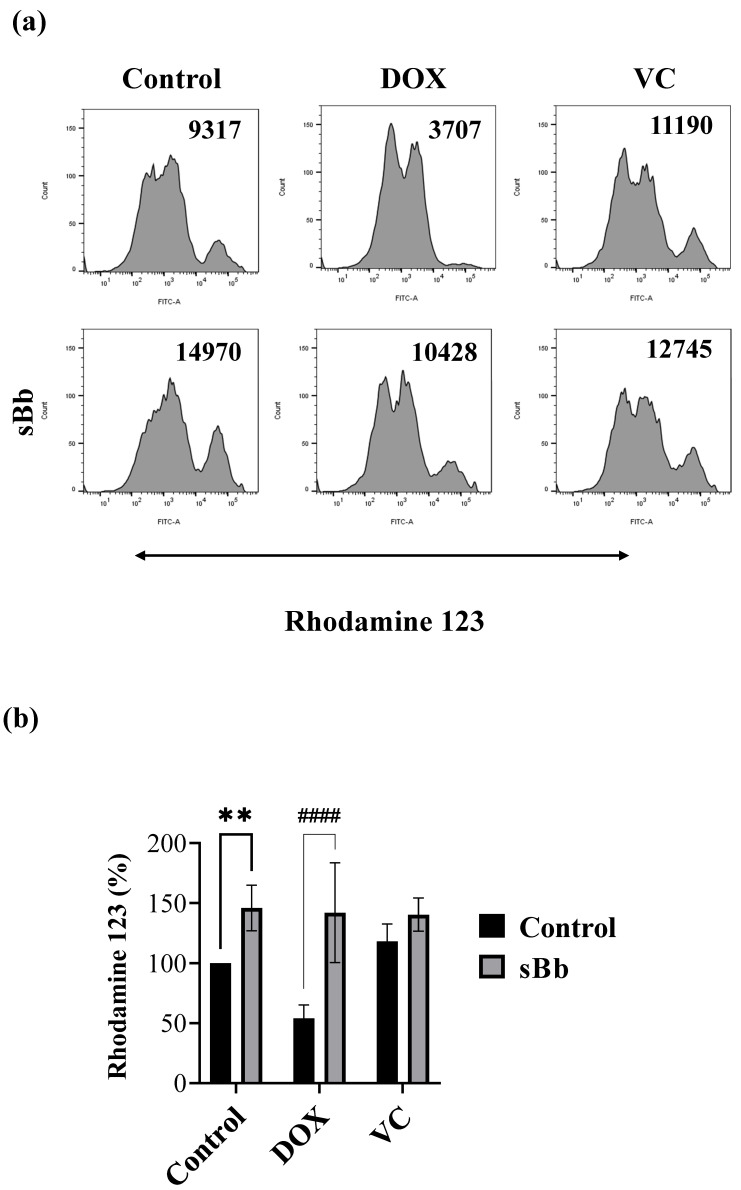
sBb increases the MMP of DOX-treated DCs. The DCs treated with DOX, VC, and/or sBb were stained with Rhodamine 123 solution and flow cytometric analysis was performed. (**a**) A representative histogram set was presented. The numbers in the upper part of the histogram indicate mean fluorescence intensity. (**b**) The graph was generated from 5 independent experiments. The mean fluorescence intensity of control DCs was set at 100%. ** indicates *p*  <  0.01 compared to control DCs, whereas #### indicates *p*  <  0.0001 compared to the treated DCs in the absence or presence of sBb.

To measure MMP, the treated DCs were stained using Rhodamine 123 solution (Figure 3). Similar to Figure 2, which presents the viability of DCs, the MMP of DOX-treated DCs was significantly lower than that of control DCs. sBb significantly increased the MMP of control DCs and DOX-treated DCs, but not that of VC-treated DCs. Interestingly, the MMP of VC-treated DCs was higher than that of control DCs. This result demonstrated that sBb can restore the suppressed MMP of DOX-treated DCs.

### 3.4. sBb Differentially up-Regulates the Expression of Surface Markers on DCs

**Figure 4:**
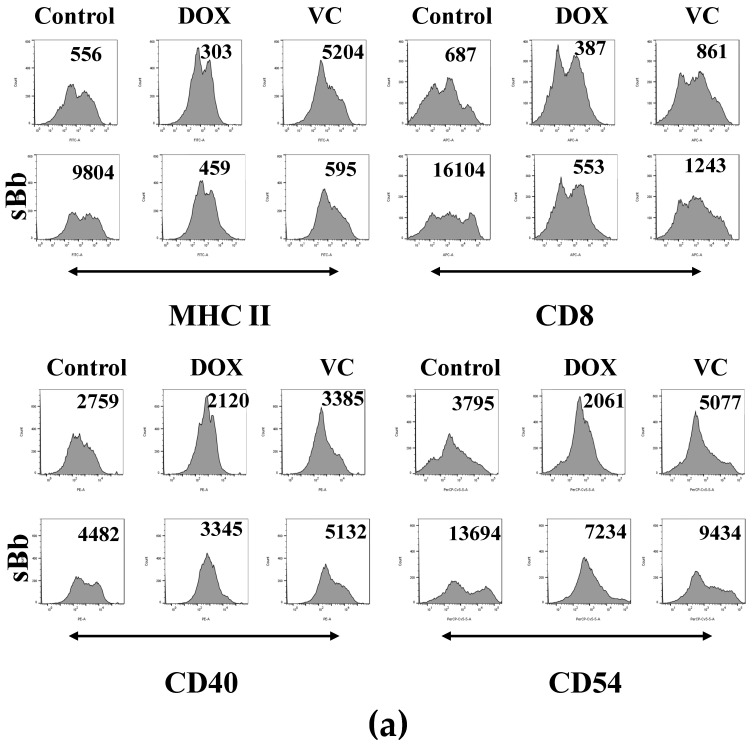

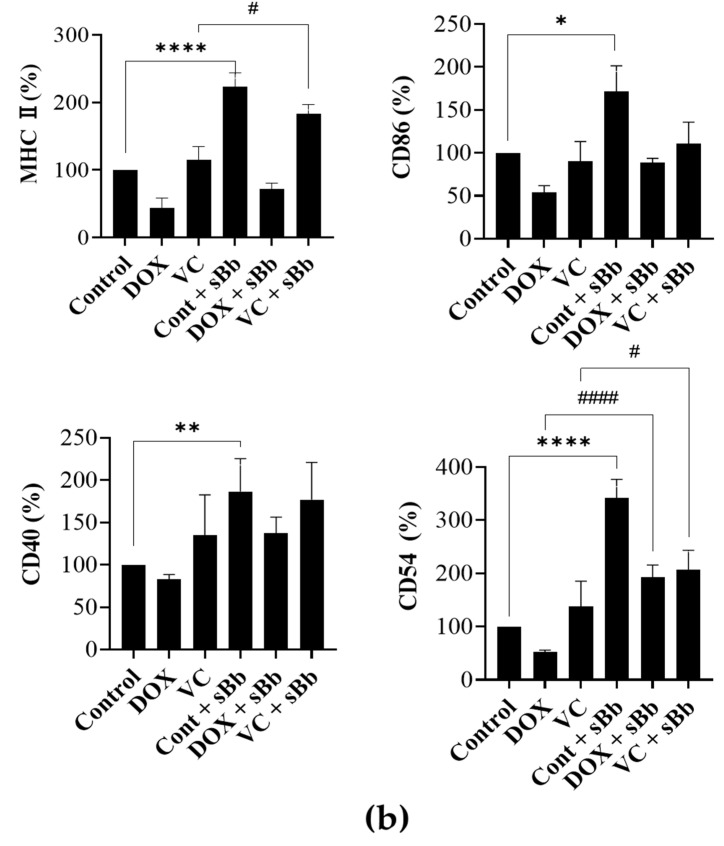
sBb differentially up-regulates the expression of surface markers on DCs. The DCs treated with DOX, VC, and/or sBb were stained with specific antibodies in the presence of Fc blocker and then analyzed by flow cytometry, as described in the Materials and Methods. (**a**) A representative histogram set was presented. Numbers in the upper part of histograms indicate mean fluorescence intensity. (**b**) The graph was generated from 3 independent experiments. The mean fluorescence intensity of control DCs was set at 100%. *, **, and **** indicate *p* < 0.05, 0.01, and 0.0001 compared to control DCs, whereas #, #### indicate *p* < 0.05, *p* < 0.0001 compared to the treated DCs in the absence or presence of sBb.

To measure the expression of immune response-related markers, the DCs were stained using anti-MHC II, CD40, CD54, and CD86 antibodies (Figure 4). sBb significantly up-regulated the expression of immune-related surface markers of control DCs and also the MHC II of VC-treated DCs and the CD54 of DOX-treated DCs and VC-treated DCs (Figure 4b).

### 3.5. sBb Increases the Antigen-Presenting Capability of DCs

**Figure 5:**
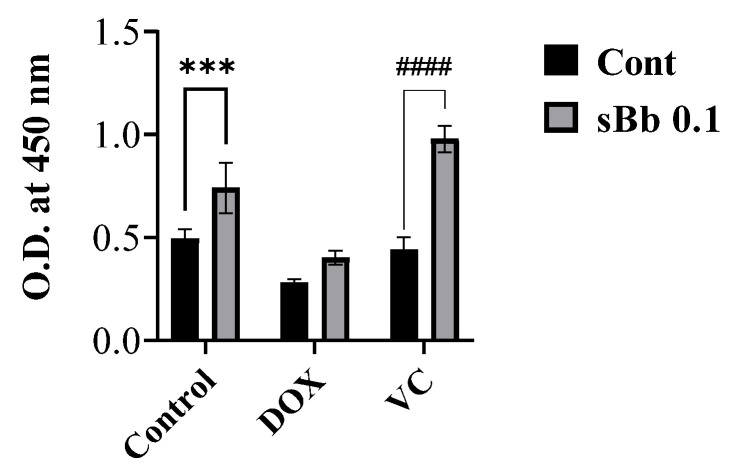
sBb increases the antigen-presenting capability of DCs. The DCs treated with DOX, VC, and/or sBb prior to co-culture and allogeneic spleen cells were co-cultured as described in the Materials and Methods. Cell Counting Kit-8 solution was used. The optical density was measured at 450 nm with a microplate reader. *** indicates *p* < 0.001 compared to control DCs, whereas #### indicates *p* < 0.0001 compared to the treated-DCs in the absence or presence of sBb.

To investigate the effects of sBb on the antigen-presenting capability of DCs, the treated DCs and allogeneic spleen cells isolated from Balb/c mice were co-cultured (Figure 5). This assay tests the allostimulatory capacity of DCs. sBb significantly increased the capability of control DCs by approximately 150% and that of VC-treated DCs by 221%. Interestingly, the VC-treated DCs showed marginally lower antigen-presenting capability than control DCs. This result demonstrated that DOX and VC differentially affected the antigen-presenting capability of DCs and that sBb can increase those of control and VC-treated DCs.

### 3.6. sBb increases the Cytokine Production of DCs

**Figure 6:**
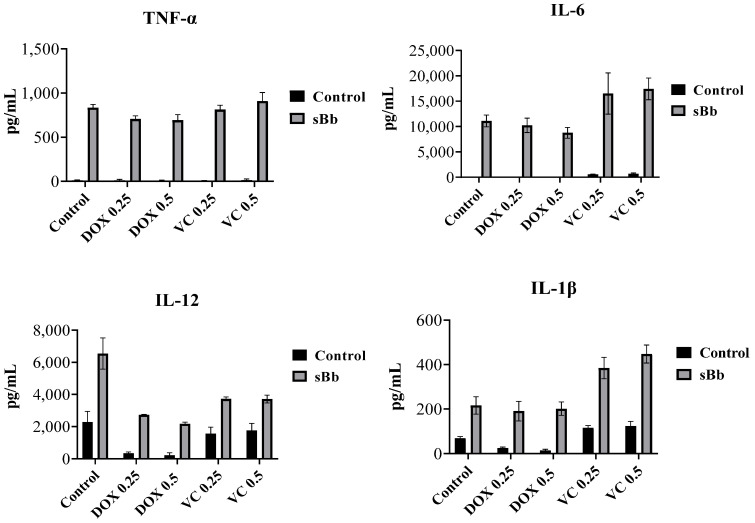
sBb increases the cytokine production of DCs. The supernatants of DOX, VC, and/or sBb treated DCs were harvested and used for ELISA. The amount of TNF-α, IL-1β, IL-6, and IL-12 produced by the treated DCs were measured by ELISA kits according to the manufacturer’s instructions.

Cytokine production was determined by ELISA (Figure 6). As a result, the DCs treated with sBb showed a marked increase in the production of all cytokines compared to control DCs. The DCs not treated with sBb produced almost no tumor necrosis factor (TNF)-α and interleukin (IL)-6. Interestingly, sBb produced more IL-1ß and IL-6 in VC-treated DCs than in other cells. DOX and VC decreased the IL-12 production of DCs compared to that of control DCs, whereas sBb recovered the reduced production to a certain level.

## 4. Discussion

We demonstrated that sBb induces the activation/maturation of DCs in various ways [18]. In this study, we conducted various experiments to study further whether sBb can protect DCs from anti-cancer drugs. Two anti-cancer drugs, DOX and VC, were used and they have different mechanisms of action and immunological characteristics. DOX acts in the cancer cells via two action mechanisms—intercalation into DNA and the generation of free radicals—leading to cell membrane breakages and DNA damage [10]. VC causes microtubule depolymerization and further inhibits chromosome separation during mitosis [11,12].

Lymphoma is one of the most commonly diagnosed cancers in dogs [8,20]. Although it can be managed with chemotherapy, only a fraction of dogs with lymphoma are completely cured, which means that it is still considered as a very challenging cancer to cure [8]. A DOX-based multi-drug protocol is considered the most effective treatment for lymphoma. To be specific, DOX, VC, cyclophosphamide, and prednisolone (CHOP) have the longest response durations and the highest response rates in the treatment of lymphoma [8].

In this study, we selected the concentrations of DOX and VC as 0.25 and 0.5 μg/mL for both drugs in our experiments. In Figure 1, the two concentrations of DOX damaged the DCs but did not kill them. Interestingly, the same concentrations of VC did not significantly damage the DCs. sBb was used at 0.1 μg/mL, a concentration that had immunostimulatory activity without damaging the DCs in a previous study [18]. In addition, to clarify the toxicity of DOX and VC we used in this study, we confirmed that DOX and VC demonstrated marked anti-cancer activity on EL-4 cells, a mouse lymphoma cell line in a concentration-dependent manner (data not shown). Furthermore, we additionally performed a MTT assay using HL60 (human leukemia cell line) and EL-4 (murine T cell lymphocyte) to make it clear whether sBb can affect the proliferation of cancer cells and confirmed that there was little or no effect of sBb on the proliferation of cancer cells (data not shown).

Based on the MTT assay and MMP analysis, sBb significantly increased the metabolic activity and viability of DCs (Figure 1, Figure 2 and Figure 3). DOX significantly damaged the DCs, whereas VC did not. The differential effect of two anti-cancer drugs on the DCs seems to come from different action mechanisms: DNA damage by the production of reactive oxygen species and blocking the depolymerization of microtubules. Previous studies have demonstrated that the anti-microtubule anti-cancer drugs, Paclitaxel and VC, at low concentrations (5 nM, 1 nM, respectively) could induce the maturation of DCs and enhance the antitumor efficacy of DCs [4]. Even though we used a relatively high concentration of VC in this study (0.25 or 0.5 μg/mL), it appears to be involved in the increase in activation/maturation of the DCs, as shown in Figure 3a and Figure 5.

To investigate the functional changes in DCs, we analyzed the expression of immune response-related surface markers, cytokine production, and antigen-presenting capability of the DCs. sBb enhanced the function of the DCs treated with DOX or VC. According to flow cytometry analysis, VC did not down-regulate MHC II, CD40, and CD54 molecules on DCs compared to control DCs. Moreover, VC + sBb showed additive effects on the expression of MHC II and CD54 of DCs (Figure 4). To investigate the production of cytokines, we selected four cytokines (TNF-α, IL-1β, IL-6, IL-12) with different functions and performed an ELISA. TNF-α and IL-1β are pro-inflammatory cytokines that induce DC maturation by the up-regulation of co-stimulatory molecules [21]. IL-6 displays both pro- and anti-inflammatory effects and is involved in the pathogenesis of many immunological diseases [22]. IL-12 is crucial to the differentiation and expansion of Th1 cells [23]. The ELISA demonstrated that sBb significantly increased the cytokine production of DCs. It seems that sBb induces the activation and maturation of DCs via cytokine production. In particular, sBb produced more IL-1ß and IL-6 in the VC-treated DCs than in other cells. This result suggests that the change in cytokine production pattern by sBb may cause alterations in the related immune responses, including Th cell differentiation. More research is needed in the future.

Taken together, we verified that sBb protects DCs from anti-cancer drug-induced damage. Anti-cancer drugs generate serious side effects during the treatment of cancers including lymphoma, especially immunosuppression [7,9,24]. To reduce these side effects, a variety of chemotherapy regimens combined with DCs have been tested [25]. It was confirmed in this study that sBb plays an important role in preventing the decrease in the viability and functional impairment of DCs that can be used for combined therapy with anti-cancer drugs. Thus this suggests that sBb has potential as an immunostimulating and protective agent that can be used as an adjuvant with anti-cancer drugs.

## Data Availability

Not applicable.
